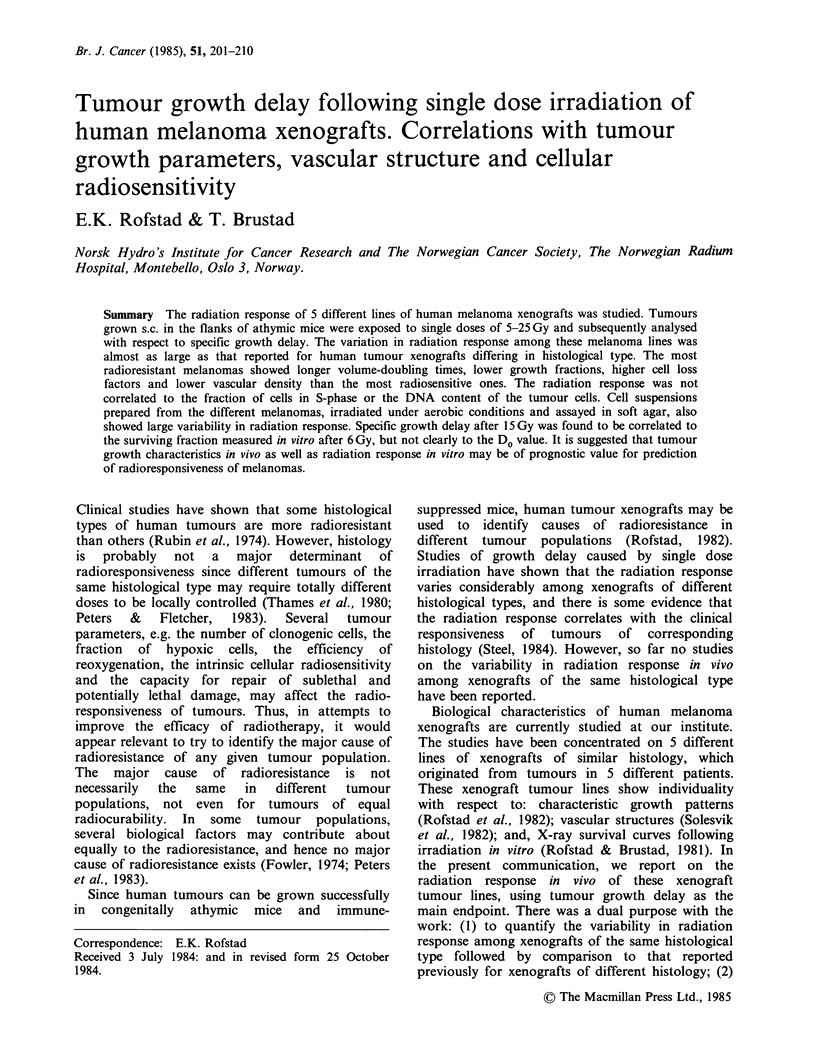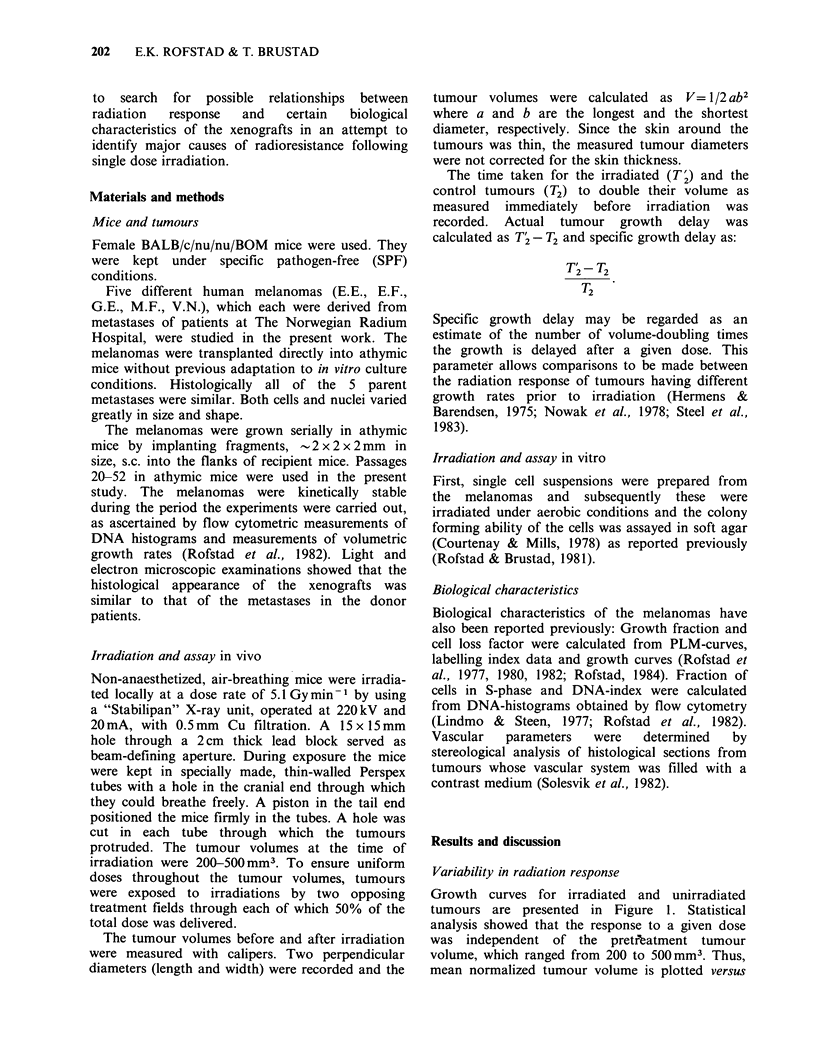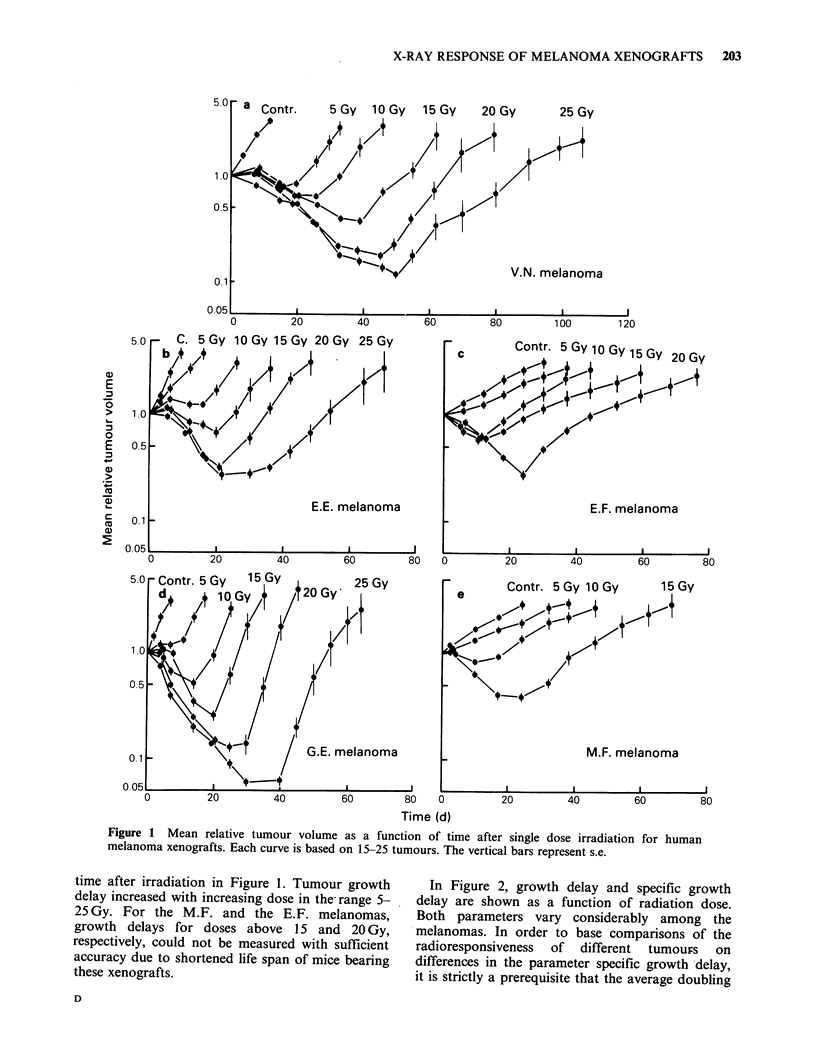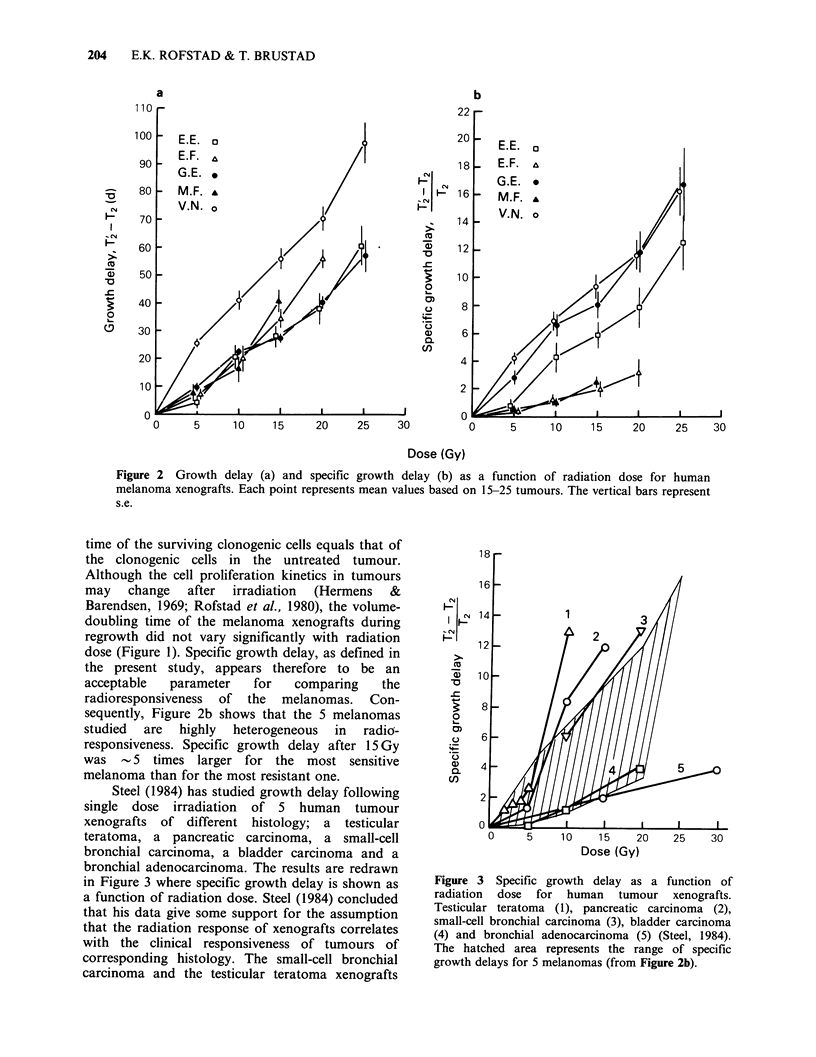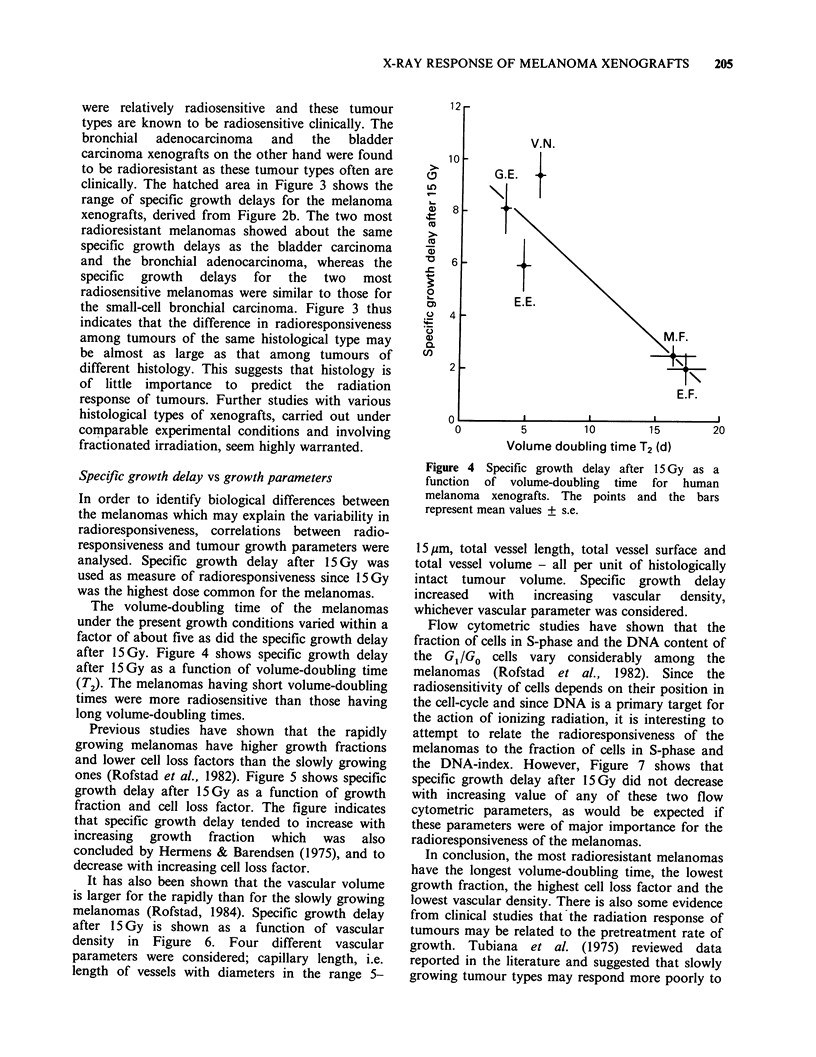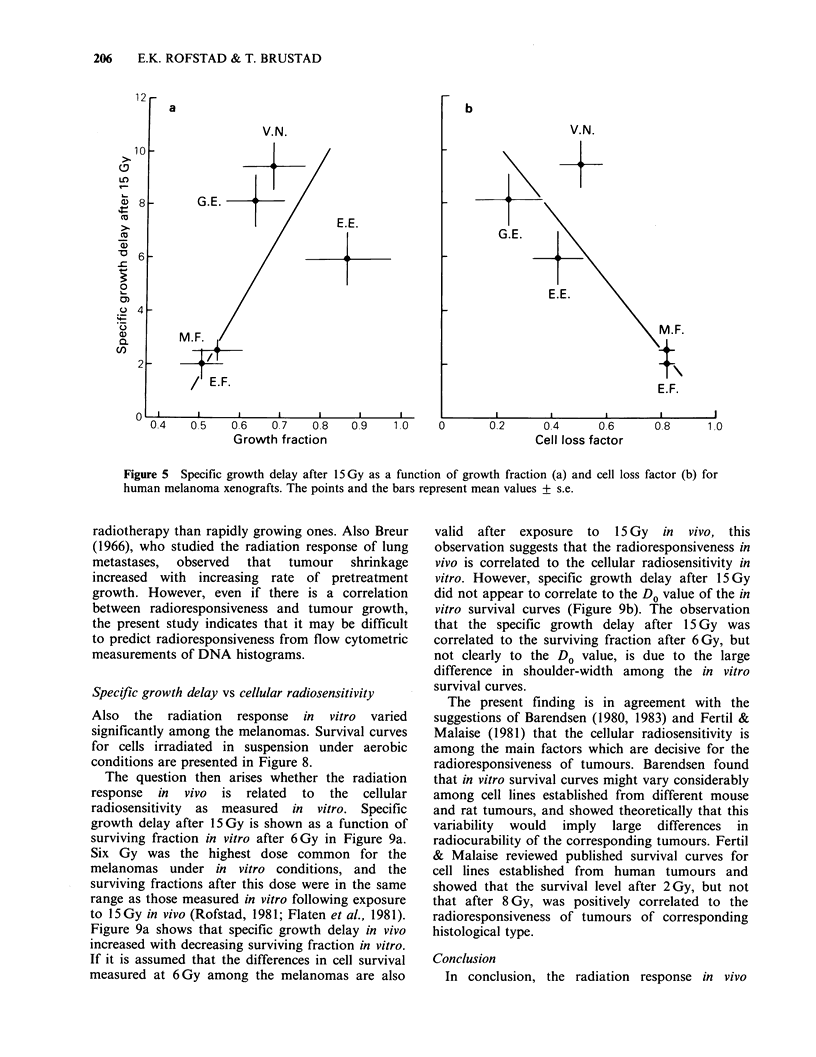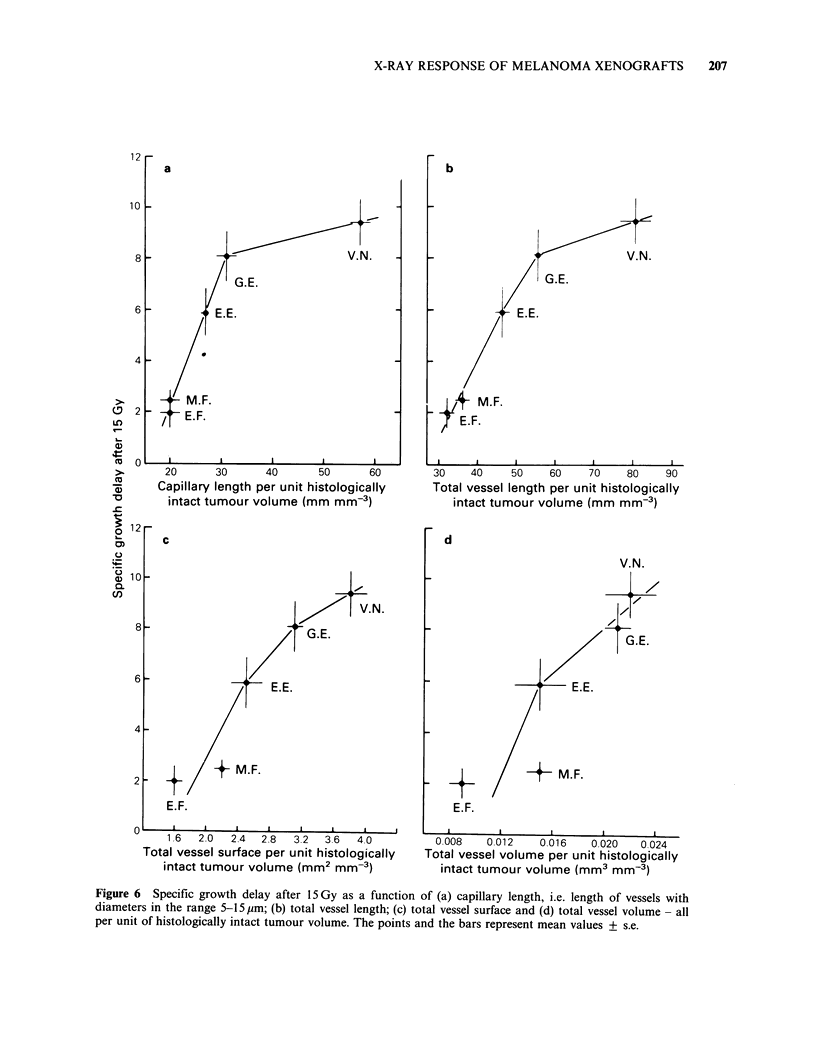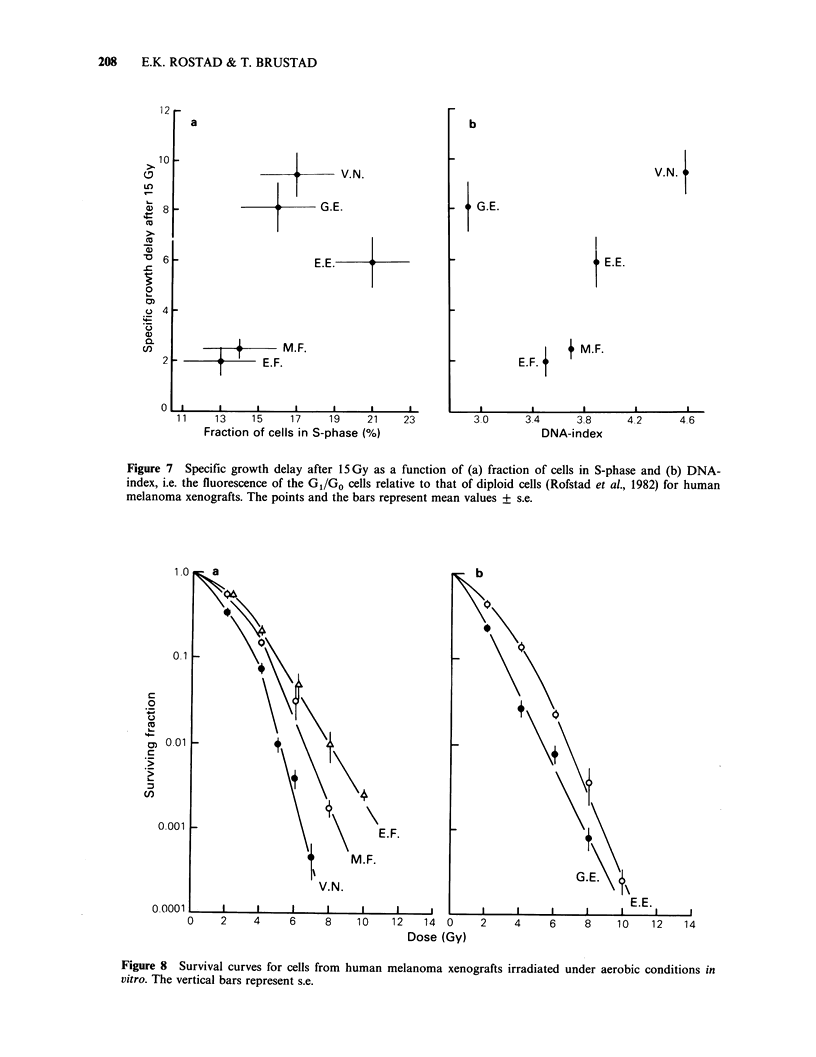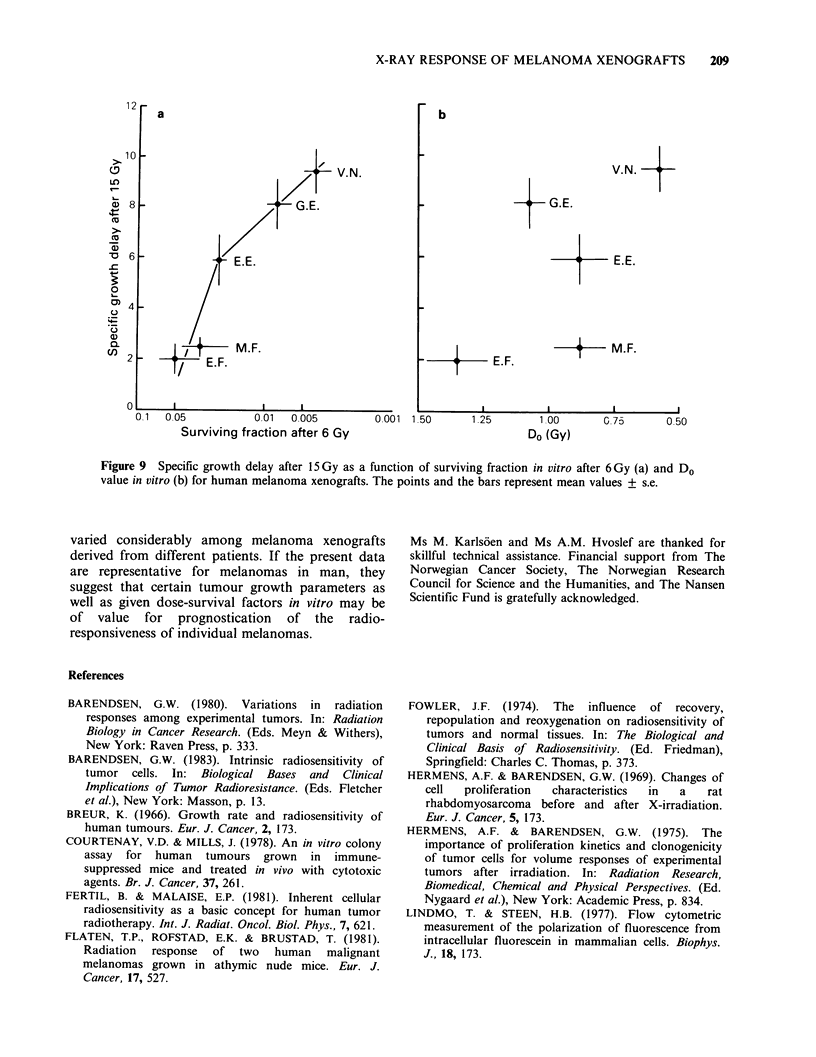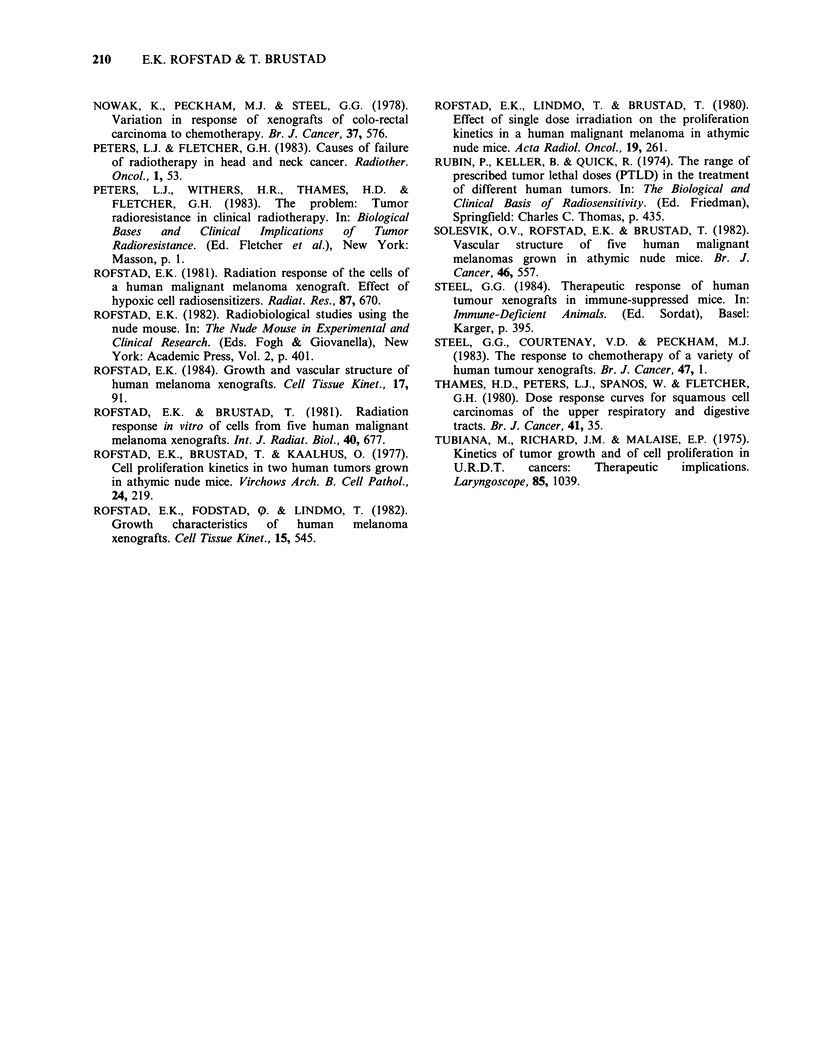# Tumour growth delay following single dose irradiation of human melanoma xenografts. Correlations with tumour growth parameters, vascular structure and cellular radiosensitivity.

**DOI:** 10.1038/bjc.1985.30

**Published:** 1985-02

**Authors:** E. K. Rofstad, T. Brustad

## Abstract

The radiation response of 5 different lines of human melanoma xenografts was studied. Tumours grown s.c. in the flanks of athymic mice were exposed to single doses of 5-25 Gy and subsequently analysed with respect to specific growth delay. The variation in radiation response among these melanoma lines was almost as large as that reported for human tumour xenografts differing in histological type. The most radioresistant melanomas showed longer volume-doubling times, lower growth fractions, higher cell loss factors and lower vascular density than the most radiosensitive ones. The radiation response was not correlated to the fraction of cells in S-phase or the DNA content of the tumour cells. Cell suspensions prepared from the different melanomas, irradiated under aerobic conditions and assayed in soft agar, also showed large variability in radiation response. Specific growth delay after 15 Gy was found to be correlated to the surviving fraction measured in vitro after 6 Gy, but not clearly to the Do value. It is suggested that tumour growth characteristics in vivo as well as radiation response in vitro may be of prognostic value for prediction of radioresponsiveness of melanomas.


*Br. J. Cancer* (1985), **51**, 201–210

### Tumour growth delay following single dose irradiation of human melanoma xenografts. Correlations with tumour growth parameters, vascular structure and cellular radiosensitivity

E.K. Rofstad & T. Brustad

*Norsk Hydro's Institute for Cancer Research and The Norwegian Cancer Society, The Norwegian Radium Hospital, Montebello, Oslo 3, Norway.*

**Summary** The radiation response of 5 different lines of human melanoma xenografts was studied. Tumours grown s.c. in the flanks of athymic mice were exposed to single doses of 5–25 Gy and subsequently analysed with respect to specific growth delay. The variation in radiation response among these melanoma lines was almost as large as that reported for human tumour xenografts differing in histological type. The most radioresistant melanomas showed longer volume-doubling times, lower growth fractions, higher cell loss factors and lower vascular density than the most radiosensitive ones. The radiation response was not correlated to the fraction of cells in S-phase or the DNA content of the tumour cells. Cell suspensions prepared from the different melanomas, irradiated under aerobic conditions and assayed in soft agar, also showed large variability in radiation response. Specific growth delay after 15 Gy was found to be correlated to the surviving fraction measured *in vitro* after 6 Gy, but not clearly to the $D_0$ value. It is suggested that tumour growth characteristics *in vivo* as well as radiation response *in vitro* may be of prognostic value for prediction of radioresponsiveness of melanomas.

Clinical studies have shown that some histological types of human tumours are more radioresistant than others (Rubin *et al.*, 1974). However, histology is probably not a major determinant of radioresponsiveness since different tumours of the same histological type may require totally different doses to be locally controlled (Thames *et al.*, 1980; Peters & Fletcher, 1983). Several tumour parameters, e.g. the number of clonogenic cells, the fraction of hypoxic cells, the efficiency of reoxygenation, the intrinsic cellular radiosensitivity and the capacity for repair of sublethal and potentially lethal damage, may affect the radioresponsiveness of tumours. Thus, in attempts to improve the efficacy of radiotherapy, it would appear relevant to try to identify the major cause of radioresistance of any given tumour population. The major cause of radioresistance is not necessarily the same in different tumour populations, not even for tumours of equal radiocurability. In some tumour populations, several biological factors may contribute about equally to the radioresistance, and hence no major cause of radioresistance exists (Fowler, 1974; Peters *et al.*, 1983).

Since human tumours can be grown successfully in congenitally athymic mice and immune-suppressed mice, human tumour xenografts may be used to identify causes of radioresistance in different tumour populations (Rofstad, 1982). Studies of growth delay caused by single dose irradiation have shown that the radiation response varies considerably among xenografts of different histological types, and there is some evidence that the radiation response correlates with the clinical responsiveness of tumours of corresponding histology (Steel, 1984). However, so far no studies on the variability in radiation response *in vivo* among xenografts of the same histological type have been reported.

Biological characteristics of human melanoma xenografts are currently studied at our institute. The studies have been concentrated on 5 different lines of xenografts of similar histology, which originated from tumours in 5 different patients. These xenograft tumour lines show individuality with respect to: characteristic growth patterns (Rofstad *et al.*, 1982); vascular structures (Solesvik *et al.*, 1982); and, X-ray survival curves following irradiation *in vitro* (Rofstad & Brustad, 1981). In the present communication, we report on the radiation response *in vivo* of these xenograft tumour lines, using tumour growth delay as the main endpoint. There was a dual purpose with the work: (1) to quantify the variability in radiation response among xenografts of the same histological type followed by comparison to that reported previously for xenografts of different histology; (2)

Correspondence: E.K. Rofstad
Received 3 July 1984: and in revised form 25 October 1984.

© The Macmillan Press Ltd., 1985

to search for possible relationships between radiation response and certain biological characteristics of the xenografts in an attempt to identify major causes of radioresistance following single dose irradiation.

### Materials and methods

### *Mice and tumours*

Female BALB/c/nu/nu/BOM mice were used. They were kept under specific pathogen-free (SPF) conditions.

Five different human melanomas (E.E., E.F., G.E., M.F., V.N.), which each were derived from metastases of patients at The Norwegian Radium Hospital, were studied in the present work. The melanomas were transplanted directly into athymic mice without previous adaptation to *in vitro* culture conditions. Histologically all of the 5 parent metastases were similar. Both cells and nuclei varied greatly in size and shape.

The melanomas were grown serially in athymic mice by implanting fragments, $\sim 2\times 2\times 2$ mm in size, s.c. into the flanks of recipient mice. Passages 20–52 in athymic mice were used in the present study. The melanomas were kinetically stable during the period the experiments were carried out, as ascertained by flow cytometric measurements of DNA histograms and measurements of volumetric growth rates (Rofstad *et al.*, 1982). Light and electron microscopic examinations showed that the histological appearance of the xenografts was similar to that of the metastases in the donor patients.

### *Irradiation and assay* in vivo

Non-anaesthetized, air-breathing mice were irradiated locally at a dose rate of 5.1 Gy min$^{-1}$ by using a "Stabilipan" X-ray unit, operated at 220 kV and 20 mA, with 0.5 mm Cu filtration. A $15\times 15$ mm hole through a 2 cm thick lead block served as beam-defining aperture. During exposure the mice were kept in specially made, thin-walled Perspex tubes with a hole in the cranial end through which they could breathe freely. A piston in the tail end positioned the mice firmly in the tubes. A hole was cut in each tube through which the tumours protruded. The tumour volumes at the time of irradiation were 200–500 mm$^3$. To ensure uniform doses throughout the tumour volumes, tumours were exposed to irradiations by two opposing treatment fields through each of which 50% of the total dose was delivered.

The tumour volumes before and after irradiation were measured with calipers. Two perpendicular diameters (length and width) were recorded and the tumour volumes were calculated as $V=1/2\,ab^2$ where $a$ and $b$ are the longest and the shortest diameter, respectively. Since the skin around the tumours was thin, the measured tumour diameters were not corrected for the skin thickness.

The time taken for the irradiated ($T'_2$) and the control tumours ($T_2$) to double their volume as measured immediately before irradiation was recorded. Actual tumour growth delay was calculated as $T'_2-T_2$ and specific growth delay as:

$$\frac{T'_2-T_2}{T_2}.$$

Specific growth delay may be regarded as an estimate of the number of volume-doubling times the growth is delayed after a given dose. This parameter allows comparisons to be made between the radiation response of tumours having different growth rates prior to irradiation (Hermens & Barendsen, 1975; Nowak *et al.*, 1978; Steel *et al.*, 1983).

### *Irradiation and assay* in vitro

First, single cell suspensions were prepared from the melanomas and subsequently these were irradiated under aerobic conditions and the colony forming ability of the cells was assayed in soft agar (Courtenay & Mills, 1978) as reported previously (Rofstad & Brustad, 1981).

### *Biological characteristics*

Biological characteristics of the melanomas have also been reported previously: Growth fraction and cell loss factor were calculated from PLM-curves, labelling index data and growth curves (Rofstad *et al.*, 1977, 1980, 1982; Rofstad, 1984). Fraction of cells in S-phase and DNA-index were calculated from DNA-histograms obtained by flow cytometry (Lindmo & Steen, 1977; Rofstad *et al.*, 1982). Vascular parameters were determined by stereological analysis of histological sections from tumours whose vascular system was filled with a contrast medium (Solesvik *et al.*, 1982).

### Results and discussion

### *Variability in radiation response*

Growth curves for irradiated and unirradiated tumours are presented in Figure 1. Statistical analysis showed that the response to a given dose was independent of the pretreatment tumour volume, which ranged from 200 to 500 mm$^3$. Thus, mean normalized tumour volume is plotted *versus*

**Figure 1** Mean relative tumour volume as a function of time after single dose irradiation for human melanoma xenografts. Each curve is based on 15–25 tumours. The vertical bars represent s.e.

time after irradiation in Figure 1. Tumour growth delay increased with increasing dose in the range 5–25 Gy. For the M.F. and the E.F. melanomas, growth delays for doses above 15 and 20 Gy, respectively, could not be measured with sufficient accuracy due to shortened life span of mice bearing these xenografts.

In Figure 2, growth delay and specific growth delay are shown as a function of radiation dose. Both parameters vary considerably among the melanomas. In order to base comparisons of the radioresponsiveness of different tumours on differences in the parameter specific growth delay, it is strictly a prerequisite that the average doubling

**Figure 2** Growth delay (a) and specific growth delay (b) as a function of radiation dose for human melanoma xenografts. Each point represents mean values based on 15–25 tumours. The vertical bars represent s.e.

time of the surviving clonogenic cells equals that of the clonogenic cells in the untreated tumour. Although the cell proliferation kinetics in tumours may change after irradiation (Hermens & Barendsen, 1969; Rofstad *et al.*, 1980), the volume-doubling time of the melanoma xenografts during regrowth did not vary significantly with radiation dose (Figure 1). Specific growth delay, as defined in the present study, appears therefore to be an acceptable parameter for comparing the radioresponsiveness of the melanomas. Consequently, Figure 2b shows that the 5 melanomas studied are highly heterogeneous in radioresponsiveness. Specific growth delay after 15 Gy was ~5 times larger for the most sensitive melanoma than for the most resistant one.

Steel (1984) has studied growth delay following single dose irradiation of 5 human tumour xenografts of different histology; a testicular teratoma, a pancreatic carcinoma, a small-cell bronchial carcinoma, a bladder carcinoma and a bronchial adenocarcinoma. The results are redrawn in Figure 3 where specific growth delay is shown as a function of radiation dose. Steel (1984) concluded that his data give some support for the assumption that the radiation response of xenografts correlates with the clinical responsiveness of tumours of corresponding histology. The small-cell bronchial carcinoma and the testicular teratoma xenografts

**Figure 3** Specific growth delay as a function of radiation dose for human tumour xenografts. Testicular teratoma (1), pancreatic carcinoma (2), small-cell bronchial carcinoma (3), bladder carcinoma (4) and bronchial adenocarcinoma (5) (Steel, 1984). The hatched area represents the range of specific growth delays for 5 melanomas (from **Figure 2b**).

were relatively radiosensitive and these tumour types are known to be radiosensitive clinically. The bronchial adenocarcinoma and the bladder carcinoma xenografts on the other hand were found to be radioresistant as these tumour types often are clinically. The hatched area in Figure 3 shows the range of specific growth delays for the melanoma xenografts, derived from Figure 2b. The two most radioresistant melanomas showed about the same specific growth delays as the bladder carcinoma and the bronchial adenocarcinoma, whereas the specific growth delays for the two most radiosensitive melanomas were similar to those for the small-cell bronchial carcinoma. Figure 3 thus indicates that the difference in radioresponsiveness among tumours of the same histological type may be almost as large as that among tumours of different histology. This suggests that histology is of little importance to predict the radiation response of tumours. Further studies with various histological types of xenografts, carried out under comparable experimental conditions and involving fractionated irradiation, seem highly warranted.

*Specific growth delay* vs *growth parameters*

In order to identify biological differences between the melanomas which may explain the variability in radioresponsiveness, correlations between radioresponsiveness and tumour growth parameters were analysed. Specific growth delay after 15 Gy was used as measure of radioresponsiveness since 15 Gy was the highest dose common for the melanomas.

The volume-doubling time of the melanomas under the present growth conditions varied within a factor of about five as did the specific growth delay after 15 Gy. Figure 4 shows specific growth delay after 15 Gy as a function of volume-doubling time ($T_2$). The melanomas having short volume-doubling times were more radiosensitive than those having long volume-doubling times.

Previous studies have shown that the rapidly growing melanomas have higher growth fractions and lower cell loss factors than the slowly growing ones (Rofstad *et al.*, 1982). Figure 5 shows specific growth delay after 15 Gy as a function of growth fraction and cell loss factor. The figure indicates that specific growth delay tended to increase with increasing growth fraction which was also concluded by Hermens & Barendsen (1975), and to decrease with increasing cell loss factor.

It has also been shown that the vascular volume is larger for the rapidly than for the slowly growing melanomas (Rofstad, 1984). Specific growth delay after 15 Gy is shown as a function of vascular density in Figure 6. Four different vascular parameters were considered; capillary length, i.e. length of vessels with diameters in the range 5–15 $\mu$m, total vessel length, total vessel surface and total vessel volume – all per unit of histologically intact tumour volume. Specific growth delay increased with increasing vascular density, whichever vascular parameter was considered.

**Figure 4** Specific growth delay after 15 Gy as a function of volume-doubling time for human melanoma xenografts. The points and the bars represent mean values ± s.e.

Flow cytometric studies have shown that the fraction of cells in S-phase and the DNA content of the $G_1/G_0$ cells vary considerably among the melanomas (Rofstad *et al.*, 1982). Since the radiosensitivity of cells depends on their position in the cell-cycle and since DNA is a primary target for the action of ionizing radiation, it is interesting to attempt to relate the radioresponsiveness of the melanomas to the fraction of cells in S-phase and the DNA-index. However, Figure 7 shows that specific growth delay after 15 Gy did not decrease with increasing value of any of these two flow cytometric parameters, as would be expected if these parameters were of major importance for the radioresponsiveness of the melanomas.

In conclusion, the most radioresistant melanomas have the longest volume-doubling time, the lowest growth fraction, the highest cell loss factor and the lowest vascular density. There is also some evidence from clinical studies that the radiation response of tumours may be related to the pretreatment rate of growth. Tubiana *et al.* (1975) reviewed data reported in the literature and suggested that slowly growing tumour types may respond more poorly to

**Figure 5** Specific growth delay after 15 Gy as a function of growth fraction (a) and cell loss factor (b) for human melanoma xenografts. The points and the bars represent mean values ± s.e.

radiotherapy than rapidly growing ones. Also Breur (1966), who studied the radiation response of lung metastases, observed that tumour shrinkage increased with increasing rate of pretreatment growth. However, even if there is a correlation between radioresponsiveness and tumour growth, the present study indicates that it may be difficult to predict radioresponsiveness from flow cytometric measurements of DNA histograms.

### *Specific growth delay* vs *cellular radiosensitivity*

Also the radiation response *in vitro* varied significantly among the melanomas. Survival curves for cells irradiated in suspension under aerobic conditions are presented in Figure 8.

The question then arises whether the radiation response *in vivo* is related to the cellular radiosensitivity as measured *in vitro*. Specific growth delay after 15 Gy is shown as a function of surviving fraction *in vitro* after 6 Gy in Figure 9a. Six Gy was the highest dose common for the melanomas under *in vitro* conditions, and the surviving fractions after this dose were in the same range as those measured *in vitro* following exposure to 15 Gy *in vivo* (Rofstad, 1981; Flaten *et al.*, 1981). Figure 9a shows that specific growth delay *in vivo* increased with decreasing surviving fraction *in vitro*. If it is assumed that the differences in cell survival measured at 6 Gy among the melanomas are also valid after exposure to 15 Gy *in vivo,* this observation suggests that the radioresponsiveness *in vivo* is correlated to the cellular radiosensitivity *in vitro*. However, specific growth delay after 15 Gy did not appear to correlate to the $D_0$ value of the *in vitro* survival curves (Figure 9b). The observation that the specific growth delay after 15 Gy was correlated to the surviving fraction after 6 Gy, but not clearly to the $D_0$ value, is due to the large difference in shoulder-width among the *in vitro* survival curves.

The present finding is in agreement with the suggestions of Barendsen (1980, 1983) and Fertil & Malaise (1981) that the cellular radiosensitivity is among the main factors which are decisive for the radioresponsiveness of tumours. Barendsen found that *in vitro* survival curves might vary considerably among cell lines established from different mouse and rat tumours, and showed theoretically that this variability would imply large differences in radiocurability of the corresponding tumours. Fertil & Malaise reviewed published survival curves for cell lines established from human tumours and showed that the survival level after 2 Gy, but not that after 8 Gy, was positively correlated to the radioresponsiveness of tumours of corresponding histological type.

### *Conclusion*

In conclusion, the radiation response *in vivo*

**Figure 6** Specific growth delay after 15 Gy as a function of (a) capillary length, i.e. length of vessels with diameters in the range 5–15 $\mu$m; (b) total vessel length; (c) total vessel surface and (d) total vessel volume – all per unit of histologically intact tumour volume. The points and the bars represent mean values ± s.e.

**Figure 7** Specific growth delay after 15 Gy as a function of (a) fraction of cells in S-phase and (b) DNA-index, i.e. the fluorescence of the $G_1/G_0$ cells relative to that of diploid cells (Rofstad *et al.*, 1982) for human melanoma xenografts. The points and the bars represent mean values ± s.e.

**Figure 8** Survival curves for cells from human melanoma xenografts irradiated under aerobic conditions *in vitro*. The vertical bars represent s.e.

**Figure 9** Specific growth delay after 15 Gy as a function of surviving fraction *in vitro* after 6 Gy (a) and $D_0$ value *in vitro* (b) for human melanoma xenografts. The points and the bars represent mean values ± s.e.

varied considerably among melanoma xenografts derived from different patients. If the present data are representative for melanomas in man, they suggest that certain tumour growth parameters as well as given dose-survival factors *in vitro* may be of value for prognostication of the radioresponsiveness of individual melanomas.

Ms M. Karlsöen and Ms A.M. Hvoslef are thanked for skillful technical assistance. Financial support from The Norwegian Cancer Society, The Norwegian Research Council for Science and the Humanities, and The Nansen Scientific Fund is gratefully acknowledged.

### References

BARENDSEN, G.W. (1980). Variations in radiation responses among experimental tumors. In: *Radiation Biology in Cancer Research.* (Eds. Meyn & Withers), New York: Raven Press, p. 333.

BARENDSEN, G.W. (1983). Intrinsic radiosensitivity of tumor cells. In: *Biological Bases and Clinical Implications of Tumor Radioresistance.* (Eds. Fletcher *et al.*), New York: Masson, p. 13.

BREUR, K. (1966). Growth rate and radiosensitivity of human tumours. *Eur. J. Cancer,* **2,** 173.

COURTENAY, V.D. & MILLS, J. (1978). An *in vitro* colony assay for human tumours grown in immune-suppressed mice and treated *in vivo* with cytotoxic agents. *Br. J. Cancer,* **37,** 261.

FERTIL, B. & MALAISE, E.P. (1981). Inherent cellular radiosensitivity as a basic concept for human tumor radiotherapy. *Int. J. Radiat. Oncol. Biol. Phys.,* **7,** 621.

FLATEN, T.P., ROFSTAD, E.K. & BRUSTAD, T. (1981). Radiation response of two human malignant melanomas grown in athymic nude mice. *Eur. J. Cancer,* **17,** 527.

FOWLER, J.F. (1974). The influence of recovery, repopulation and reoxygenation on radiosensitivity of tumors and normal tissues. In: *The Biological and Clinical Basis of Radiosensitivity.* (Ed. Friedman), Springfield: Charles C. Thomas, p. 373.

HERMENS, A.F. & BARENDSEN, G.W. (1969). Changes of cell proliferation characteristics in a rat rhabdomyosarcoma before and after X-irradiation. *Eur. J. Cancer,* **5,** 173.

HERMENS, A.F. & BARENDSEN, G.W. (1975). The importance of proliferation kinetics and clonogenicity of tumor cells for volume responses of experimental tumors after irradiation. In: *Radiation Research, Biomedical, Chemical and Physical Perspectives.* (Ed. Nygaard *et al.*), New York: Academic Press, p. 834.

LINDMO, T. & STEEN, H.B. (1977). Flow cytometric measurement of the polarization of fluorescence from intracellular fluorescein in mammalian cells. *Biophys. J.,* **18,** 173.

NOWAK, K., PECKHAM, M.J. & STEEL, G.G. (1978). Variation in response of xenografts of colo-rectal carcinoma to chemotherapy. *Br. J. Cancer,* **37,** 576.

PETERS, L.J. & FLETCHER, G.H. (1983). Causes of failure of radiotherapy in head and neck cancer. *Radiother. Oncol.,* **1,** 53.

PETERS, L.J., WITHERS, H.R., THAMES, H.D. & FLETCHER, G.H. (1983). The problem: Tumor radioresistance in clinical radiotherapy. In: *Biological Bases and Clinical Implications of Tumor Radioresistance.* (Ed. Fletcher *et al.*), New York: Masson, p. 1.

ROFSTAD, E.K. (1981). Radiation response of the cells of a human malignant melanoma xenograft. Effect of hypoxic cell radiosensitizers. *Radiat. Res.,* **87,** 670.

ROFSTAD, E.K. (1982). Radiobiological studies using the nude mouse. In: *The Nude Mouse in Experimental and Clinical Research.* (Eds. Fogh & Giovanella), New York: Academic Press, Vol. 2, p. 401.

ROFSTAD, E.K. (1984). Growth and vascular structure of human melanoma xenografts. *Cell Tissue Kinet.,* **17,** 91.

ROFSTAD, E.K. & BRUSTAD, T. (1981). Radiation response *in vitro* of cells from five human malignant melanoma xenografts. *Int. J. Radiat. Biol.,* **40,** 677.

ROFSTAD, E.K., BRUSTAD, T. & KAALHUS, O. (1977). Cell proliferation kinetics in two human tumors grown in athymic nude mice. *Virchows Arch. B. Cell Pathol.,* **24,** 219.

ROFSTAD, E.K., FODSTAD, Ø. & LINDMO, T. (1982). Growth characteristics of human melanoma xenografts. *Cell Tissue Kinet.,* **15,** 545.

ROFSTAD, E.K., LINDMO, T. & BRUSTAD, T. (1980). Effect of single dose irradiation on the proliferation kinetics in a human malignant melanoma in athymic nude mice. *Acta Radiol. Oncol.,* **19,** 261.

RUBIN, P., KELLER, B. & QUICK, R. (1974). The range of prescribed tumor lethal doses (PTLD) in the treatment of different human tumors. In: *The Biological and Clinical Basis of Radiosensitivity.* (Ed. Friedman), Springfield: Charles C. Thomas, p. 435.

SOLESVIK, O.V., ROFSTAD, E.K. & BRUSTAD, T. (1982). Vascular structure of five human malignant melanomas grown in athymic nude mice. *Br. J. Cancer,* **46,** 557.

STEEL, G.G. (1984). Therapeutic response of human tumour xenografts in immune-suppressed mice. In: *Immune-Deficient Animals.* (Ed. Sordat), Basel: Karger, p. 395.

STEEL, G.G., COURTENAY, V.D. & PECKHAM, M.J. (1983). The response to chemotherapy of a variety of human tumour xenografts. *Br. J. Cancer,* **47,** 1.

THAMES, H.D., PETERS, L.J., SPANOS, W. & FLETCHER, G.H. (1980). Dose response curves for squamous cell carcinomas of the upper respiratory and digestive tracts. *Br. J. Cancer,* **41,** 35.

TUBIANA, M., RICHARD, J.M. & MALAISE, E.P. (1975). Kinetics of tumor growth and of cell proliferation in U.R.D.T. cancers: Therapeutic implications. *Laryngoscope,* **85,** 1039.